# Highly Specific Monoclonal Antibody Targeting the Botulinum Neurotoxin Type E Exposed SNAP-25 Neoepitope

**DOI:** 10.3390/antib11010021

**Published:** 2022-03-16

**Authors:** Adva Mechaly, Eran Diamant, Ron Alcalay, Alon Ben David, Eyal Dor, Amram Torgeman, Ada Barnea, Meni Girshengorn, Lilach Levin, Eyal Epstein, Ariel Tennenhouse, Sarel J. Fleishman, Ran Zichel, Ohad Mazor

**Affiliations:** 1 Department of Infectious Diseases, Israel Institute for Biological Research, Ness-Ziona 7410001, Israel; advam@iibr.gov.il; 2 Department of Biotechnology, Israel Institute for Biological Research, Ness-Ziona 7410001, Israel; erand@iibr.gov.il (E.D.); alonb@iibr.gov.il (A.B.D.); eyalo@iibr.gov.il (E.D.); amit@iibr.gov.il (A.T.); adab@iibr.gov.il (A.B.); menig@iibr.gov.il (M.G.); lilachl@iibr.gov.il (L.L.); eyale@iibr.gov.il (E.E.); ranz@iibr.gov.il (R.Z.); 3 Department of Biochemistry and Molecular Genetics, Israel Institute for Biological Research, Ness-Ziona 7410001, Israel; rona@iibr.gov.il; 4 Department of Biomolecular Sciences, Weizmann Institute of Science, Rehovot 7600001, Israel; ariel.tennenhouse@weizmann.ac.il (A.T.); sarel.fleishman@weizmann.ac.il (S.J.F.)

**Keywords:** botulinum E, monoclonal antibody, phage-display, SNAP-25

## Abstract

Botulinum neurotoxin type E (BoNT/E), the fastest acting toxin of all BoNTs, cleaves the 25 kDa synaptosomal-associated protein (SNAP-25) in motor neurons, leading to flaccid paralysis. The specific detection and quantification of the BoNT/E-cleaved SNAP-25 neoepitope can facilitate the development of cell-based assays for the characterization of anti-BoNT/E antibody preparations. In order to isolate highly specific monoclonal antibodies suitable for the in vitro immuno-detection of the exposed neoepitope, mice and rabbits were immunized with an eight amino acid peptide composed of the C-terminus of the cleaved SNAP-25. The immunized rabbits developed a specific and robust polyclonal antibody response, whereas the immunized mice mostly demonstrated a weak antibody response that could not discriminate between the two forms of SNAP-25. An immune scFv phage-display library was constructed from the immunized rabbits and a panel of antibodies was isolated. The sequence alignment of the isolated clones revealed high similarity between both heavy and light chains with exceptionally short HCDR3 sequences. A chimeric scFv-Fc antibody was further expressed and characterized, exhibiting a selective, ultra-high affinity (pM) towards the SNAP-25 neoepitope. Moreover, this antibody enabled the sensitive detection of cleaved SNAP-25 in BoNT/E treated SiMa cells with no cross reactivity with the intact SNAP-25. Thus, by applying an immunization and selection procedure, we have isolated a novel, specific and high-affinity antibody against the BoNT/E-derived SNAP-25 neoepitope. This novel antibody can be applied in in vitro assays that determine the potency of antitoxin preparations and reduce the use of laboratory animals for these purposes.

## 1. Introduction

Botulinum neurotoxins (BoNTs), the most potent toxins known in nature, are synthesized by the anaerobic bacterium *Clostridium botulinum* [1]. The BoNT serotypes A, B, E, and, rarely, F are the causative agents of human botulism, a life-threatening disease [2]. BoNT is a single ~150 kDa polypeptide comprised of a ~100 kDa heavy chain (HC) bridged by an S–S bond to a ~50 kDa light chain (LC) which is a zinc-dependent endopeptidase [3]. The C-terminal portion of the HC (H_C_) binds specific receptors on the presynaptic nerve ending membrane of cholinergic neurons, and the N-terminus of the heavy chain (H_N_) facilitates the translocation of the LC into the cytosol, where it cleaves one of three soluble N-ethylmaleimide-sensitive factor attachment protein receptor (SNARE) proteins [4]. Serotypes A and E cleave the 25 kDa synaptosomal-associated protein (SNAP-25), serotypes B, D, F, and G cleave the vesicle-associated membrane protein (VAMP or synaptobrevin), and serotype C acts on both SNAP-25 and syntaxin [5]. Each serotype has a specific SNARE cleavage site, preventing the release of the neurotransmitter acetylcholine from nerve cells into the synapses [6,7,8]. Specifically, BoNT/E cleaves the 206 amino acid SNAP-25 protein (SNAP-25_1–206_) between Arg180 and Ile181, and as a result a truncated SNAP-25_1–180_ is formed [9,10]. BoNT/E exerts its toxicity faster and significantly earlier than BoNT/A and BoNT/B [11], requiring earlier diagnostic and medical responses.

The standard therapy for botulism in adults, including in cases of BoNT/E intoxications, involves treatment with antitoxin preparations [12,13] derived from hyperimmune horses (A human antitoxin preparation is also available for infant botulism cases involving BoNT/A or BoNT/B [14]). The potencies of both the pharmaceutical antitoxin and the toxicity of BoNT/E preparations intended for horse immunization are determined in vivo by the pharmacopeia mouse neutralization assay (PMNA) [15] and by the mouse bioassay (MBA) [16], respectively. However, these in vivo assays require a large number of laboratory animals, and hence the development of alternative in vitro methods to measure the activity of BoNT/E and the concentration of neutralizing antibodies is desirable.

Assays with the potential to replace MBA and PMNA should be based on in vitro demonstration of the three intoxication steps: receptor binding by the H_C_, internalization by the H_N_, and enzymatic activity of the LC. Several methods can be applied to simulate LC enzymatic activity in vitro using a synthetic substrate, and to further detect the resultant cleavage products by using mass spectrometry, fluorescence, or an immunoassay with specific antibodies [17,18,19,20,21,22,23]. Cell-based in vitro assays can combine the enzymatic activity step with the two earlier intoxication steps [24,25]. Such assays are based on the correlation between the BoNT concentration incubated with the target cells and the specific quantitation of toxin cleavage product detected using a specific antibody. Indeed, we have previously reported on a SiMa cell-based neutralization assay to measure the potency of anti-BoNT/A pharmaceutical antibody preparations in vitro [25]. This assay is based on a monoclonal chimeric antibody (MAb) that specifically recognizes the BoNT/A-cleaved SNAP-25 (anti SNAP-25_1–197_). Similarly, a proof-of-concept for the development of a SiMa cell-based neutralization assay to measure the activity of BoNT/E-cleaved SNAP-25 was shown before, using polyclonal antibodies [26]. Yet, in order to develop a reproducible assay, it is advisable to use well-characterized monoclonal antibodies.

To the best of our knowledge, despite the importance of developing such antibodies, only two groups have succeeded in isolating such antibodies using the traditional hybridoma cloning technique so far [27,28]. We have previously isolated a panel of potent antibodies from immunized animals by incorporating immunization methodologies that promote high affinity antibodies in vivo together with efficient screening methods using phage-display libraries [29,30,31]. We thus hypothesized that, by applying similar methodologies, we would be able to isolate high-affinity antibodies that specifically recognize the cleaved SNAP-25 (SNAP-25_1–180_) and not the intact SNAP-25_1–206_. Such antibodies could serve as the basis for sensitive in vitro neutralization assays. Here, we report the immunization strategy and selection procedures taken to reach this end and characterize the novel monoclonal antibody isolated. 

## 2. Materials and Methods

### 2.1. Peptides

All the peptides used in this work were synthesized by GenScript (Piscataway, NJ, USA), as follows: KLH-P1: peptide CTQNRQIDR conjugated to KLH; Bio-P1: biotinylated peptide TQNRQIDR (also referred to as W.T.); Bio-P2: biotinylated peptide MGNEIDTQNRQIDRIMEKAD; P3: IIGNLRHMALDMGNEIDTQNRQIDRIMEKAD; 173–179: biotinylated peptide TQNRQID; 173–181: biotinylated peptide TQNRQIDRI; 173–180 amide: biotinylated peptide TQNRQIDR-amide; R180K: biotinylated peptide TQNRQIDK; R180Q: biotinylated peptide TQNRQIDQ; R180E: biotinylated peptide TQNRQIDE; R180A: biotinylated peptide TQNRQIDA; R176A: biotinylated peptide TQNAQIDQ.

### 2.2. Animal Immunization

Experiments were approved by the IIBR Animal Care and Use Committee and were conducted in accordance with the guidelines of the care and use of laboratory animals published by the Israeli Ministry of Health (protocols RB-27-16 and M-60-16). All efforts were made to minimize animal suffering. All animals were observed for morbidity and mortality, overt signs of toxicity, and any signs of distress throughout the study.

Two female New Zealand White (NZW) rabbits (*Oryctolagus cuniculus*) and five female BALB/c mice (*Mus musculus*) were immunized with KLH-P1, a synthetic peptide corresponding to residues 173–180 of the human SNAP-25 sequence, conjugated to keyhole limpet hemocyanin (KLH). A cysteine residue, added to the N-terminus of the peptide, allowed coupling to KLH for immunization. Rabbits and mice were injected subcutaneously (s.c.) with 400 µg and 5 µg of antigen per animal, respectively, mixed either with Complete Freund’s Adjuvant (CFA) for priming or Incomplete Freund’s Adjuvant (IFA) for four booster immunizations. 

### 2.3. ScFv Library Construction and Screening

RNA from both rabbits was extracted from lymph nodes and spleen using the RNeasy mini kit and from blood samples using the RNeasy Protect Animal Blood kit (Qiagen GmbH, Hilden, Germany) according to the manufacturer′s instructions. All RNA samples were mixed together (per rabbit), and first-strand synthesis was performed using the Verso cDNA synthesis kit (Thermo Scientific, Waltham, MA, USA), with random hexamers and 1 µg RNA. VH and Vκ fragment amplification, construction of the scFv library and panning were carried out using pCC16 phagemid vector [32], as described in extensive detail [29], with the following changes: A Maxisorp 96-well microtiter plate (Nunc, Sigma-Aldrich, St. Louis, MO, USA) was coated with 5 µg/mL streptavidin (Sigma s0977) diluted in NaHCO_3_ buffer (50 mM, pH 9.6), and incubated overnight. The plate was then washed, blocked (3% BSA + 0.05% Tween 20 in PBS) and loaded with synthetic peptide Bio-P1 (2 µg/mL). After a 30-min incubation and a wash step, approximately 1 × 10^11^ pfu of blocked (3% skim milk + 0.05% Tween 20 in PBS) phage clones were incubated for 60 min with the peptide coated plate. In one of the enrichment schemes, the phage blocking buffer contained synthetic peptide P3 (5 µg/mL). The plate was then washed once with blocker solution followed by a total of six washes with PBST (PBS, 0.05% Tween 20). Bound phage clones were then eluted and used to infect 5 mL of logarithmic-phase TG1 strain *E. coli* (Lucigen, Middleton, WI, USA). Two additional panning rounds were conducted (for the 2nd and 3rd rounds, respectively) using 10^10^ and 10^9^ phage clones as input, while antigen incubation time was reduced to 30 and 15 min, phage blocking buffer was alternated (between 3% BSA to 3% Skim milk in PBS) and the PBST washing steps were raised to include 9 or 15 washes with PBST (0.1%). 

### 2.4. Production of Chimeric Antibodies

Phagemid DNA of the desired clones was isolated using QIAprep spin Miniprep kit (Qiagen, GmbH, Hilden, Germany), and the entire scFv was cloned into a mammalian immunoglobulin-based expression vector [33]. The vector was modified based on a published work [34], providing the scFv with the human constant Hc gene (IgG1), resulting in chimeric rabbit-human scFv-Fc antibody. FreeStyle Max 293 cells (Thermo Scientific, Waltham, MA, USA) were transiently transfected with the vector, and after one week the supernatant was collected and the antibodies were purified on a HiTrap Protein-A column (GE healthcare, Little Chalfont, UK). 

### 2.5. ELISA

Maxisorp 96-well microtiter plates were coated overnight with 5 µg/mL streptavidin (50 µL/well) in NaHCO_3_ buffer (50 mM, pH 9.6), then washed and blocked with PBST buffer (0.05% Tween 20, 2% BSA in PBS) for one hour. Peptides (2 µg/mL) were then loaded on the plate for 30 min. Rabbit sera, individual phage clones or purified antibodies were added to the plates for a one-hour incubation. The plates were then washed with PBST and incubated with the detecting antibody: alkaline phosphatase (AP)-conjugated goat anti rabbit for rabbit serum (Sigma-A8025), horseradish peroxidase (HRP)-conjugated anti-M13 antibody (GE healthcare, Little Chalfont, UK) for phage clones and AP-conjugated anti-human IgG (Sigma-A3187) for scFv-Fc antibodies. Detection of HRP conjugates was carried out with 3,3′,5,5′-tetramethybenzidine (TMB/E, Millipore, Billerica, MA, USA). Detection of AP-conjugated antibodies was carried out with SIGMAFAST p-nitrophenyl phosphate tablets (Sigma-N2770). 

### 2.6. Affinity and Specificity Measurements

Binding studies were carried out using the Octet Red system (ForteBio, Version 8.1, Menlo Park, CA, USA, 2015) that measures biolayer interferometry (BLI) [35]. All steps were performed at 30 °C with shaking at 1500 rpm in a black 96-well plate containing 200 µL solution in each well. Streptavidin-coated biosensors were loaded with the biotinylated peptides Bio-P1 or Bio-P2 (5 µg/mL) for 300 s, followed by a wash. The sensors were then reacted for 300 s with increasing concentrations of antibody and then moved to buffer-containing wells for another 300 s (dissociation phase). Binding and dissociation were measured as changes over time in light interference after the subtraction of parallel measurements from unloaded biosensors. Sensorgrams were fitted with a 1:1 binding model using the Octet data analysis software 8.1 (Fortebio, Menlo Park, CA, USA, 2015), and the presented values are an average of several repeated measurements. 

### 2.7. Bacteria and Toxins

*Clostridium botulinum* E and A strains were obtained from the IIBR collection (E450 and A198, respectively). Sequence analysis revealed that the neurotoxin genes were consistent with the serotypes NCTC11219 (GenBank accession number X62683) and 62A (GenBank accession number M30196) for types E and A, respectively [36,37]. The toxin complexes were prepared from the concentrated supernatants of cultures grown for six days in anaerobic culture tubes. The toxin complex of type E was used in its activated form. Activation was performed by treatment with 0.1% (wt/vol) trypsin (37 °C for 1 h). The toxin activities were calibrated by a standard mouse bioassay. The specific activities of toxin types E and A were 110.4 and 135.1 pg/LD_50_, respectively. 

### 2.8. Exposure of SiMa cells to BoNT

The human neuroblastoma cell line SiMa (ACC 164, Leibniz Institute, DSMZ—German Collection of Microorganisms and Cell Cultures GmbH, Braunschweig, Germany) was cultured (5 × 10^4^ cells/well) in differentiation medium containing serum-free Minimum Essential Medium (MEM) with Earls salts and Glutamax (Gibco, Thermo Fisher Scientific, Waltham, MA, USA), supplemented with N-2 (×1) (Gibco), B-27 (×1) (Gibco), and 25 µg/mL GT1b (Enzo, New York, NY, USA), as previously described [24,38], and differentiated for 48 h in 96-well plates coated with poly-D-lysine (Corning Inc., Corning, NY, USA). After differentiation, the medium was removed from the cells and replaced with 100 μL fresh GT1b-free differentiation medium containing 0, 400, 1000 or 4000 LD_50_/mL of BoNT/E, or 4000 LD_50_/mL of BoNT/A. Each treatment (toxin type and concentration) was conducted at least in quadruplicate. Following incubation for 24 h at 37 °C, the medium was replaced with fresh MEM, and the cells were incubated for an additional 24 h at 37 °C. The cells were then lysed by incubation with a cold lysis buffer (70 µL/well) containing 0.1% Triton X-100, 150 mM NaCl, 1.5 mM MgCl_2_, 1 mM EGTA, 50 mM HEPES, and protease inhibitor cocktail (EDTA-free cOmplete tabs, Roche Diagnostics GmbH, Manheim, Germany) in water for 30 min.

### 2.9. Western-Blot

Chimeric MAb and rabbit sera were tested for specificity by Western blot. Lysates containing equal concentrations of total protein, as measured by the Bradford assay, were loaded to a NuPAGE 10% Bis-Tris Gel (Invitrogen, Carlsbad, CA, USA), and the electrophoresed proteins were transferred to an Amersham Protran Premium 0.45 µm nitrocellulose membrane (GE Healthcare 10600096). The membrane was then blocked (1% skim milk in PBS) and probed with chimeric MAb or immunized rabbit sera. Rabbit anti-SNAP-25 polyclonal antibody raised against a synthetic peptide corresponding to the N-terminus of human SNAP-25 (Sigma S9684), was used as a positive control to detect both the cleaved and the intact SNAP-25. AP-conjugated anti-human IgG (Sigma A3187) and AP-conjugated anti-rabbit IgG (Jackson 711-066-152) were used as secondary antibodies for chimeric MAb and rabbit sera, respectively. 5-Bromo-4-Chloro-3-Indolyl-phosphate (BCIP, Sigma B8503) was used as the chromogenic substrate. Densitometry analysis showed that the total SNAP-25 (cleaved and intact) in the BoNT/E treated samples produced similar signals, further confirming that equal amounts of the subject protein were loaded to the gels (Figure S1).

### 2.10. Antibody Variable Domain Structure Prediction

Antibody variable domain model structures were generated using ColabFold [39], which is an accessible webserver for running the AlphaFold2 ab initio structure-prediction algorithm [40]. The model structures were generated using three recycles, templates, and AlphaFold-Multimer [41]. The models were ranked by predicted TM (pTM) score, and the top-ranked model was chosen.

## 3. Results

### 3.1. Immunization and Characterization of the Elicited Antibody Response

**Figure 1:**
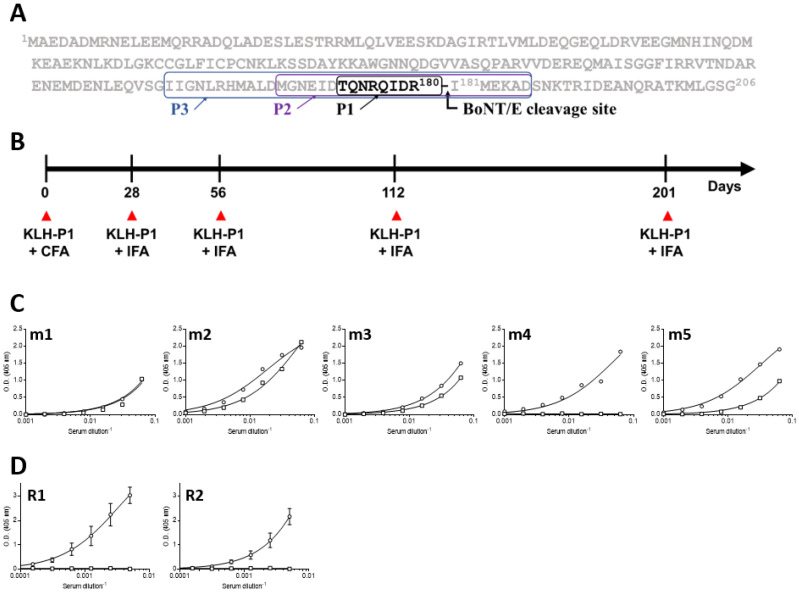
Peptide design, rabbit immunization and antibody serum levels. (**A**) SNAP-25 protein sequence highlighting the BoNT/E cleavage site and the peptides used in this study. (**B**) Animal immunization regimen using KLH-P1 in either CFA (priming) or IFA (boosting). ELISA binding curves of serum of (**C**) immunized mice or (**D**) rabbits against Bio-P1 (circles) or Bio-P2 (squares). Lines represents non-linear regression fit, and (**D**) points are the mean ± STD of duplicates.

The successful isolation of specific high affinity antibodies from phage-display immune-libraries depends largely on the effectiveness of the immunization regimen. To elicit a polyclonal antibody response that will enable the specific recognition of BoNT/E cleaved SNAP-25, mice and rabbits were immunized with an eight-residue synthetic peptide coupled to KLH via an added cysteine residue (Figure 1A), representing the resulting C-terminal end of SNAP-25 after BoNT/E activity. The immunization process included a prime injection and boosts given after 1, 2, 4 and 7 months (Figure 1B). Antibody titers were determined by ELISA against the specific synthetic peptide (Bio-P1), representing the resulting cleavage site of SNANP-25 after BoNT/E activity. Interestingly, while all immunized animals developed a significant antibody titer against the Bio-P1 peptide, the rabbits’ response was about 10-fold higher than that of the mice (Figure 1C). Specificity was determined by comparing the response on the specific peptide (Bio-P1) to the response attained on the non-specific peptide (Bio-P2), representing the non-cleaved SNAP-25 (Figure 1C). Out of the five immunized mice, only one (m4) developed a specific response. In marked contrast, the two rabbits developed a highly specific antibody response toward Bio-P1, with no cross-reactivity observed on Bio-P2. As the two rabbits’ antibody titers were similar to each other and about 10-fold higher than mouse m4, we decided to continue characterizing the rabbits’ sera.

**Figure 2:**
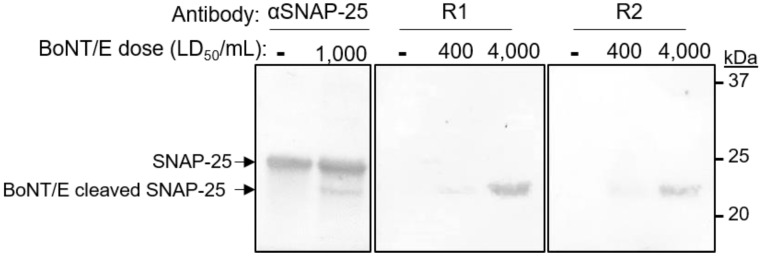
Specificity of an immunized rabbit’s sera. Differentiated SiMa cells were exposed to 0, 400 or 4000 LD_50_/mL of BoNT/E and lysed 24 h after intoxication. Lysates were subjected to SDS-PAGE and Western-blotted using either anti-SNAP-25 polyclonal antibody or Bio-P1 immunized rabbits (R1 and R2).

While specific recognition of the cleaved peptide was demonstrated, it was important to further verify that these antibodies can also specifically recognize BoNT/E-mediated cleaved SNAP-25. We previously demonstrated that SNAP-25 cleavage by BoNT/A can be monitored in intoxicated SiMa cells by using a specific antibody [25]. Here, cells were first incubated with 1000 LD_50_/mL of BoNT/E, washed and lysed 24 h later. The cell lysate was resolved on SDS-PAGE and probed with an antibody directed against the N-terminus of the human SNAP-25. As expected, this antibody recognized both the intact (25 kDa) and the cleaved (23 kDa) SNAP-25 (Figure 2). Next, the serum of each immunized rabbit was reacted with lysates of cells that were exposed to either 400 or 4000 LD_50_/mL of BoNT/E. Indeed, the sera of both animals specifically recognized only the cleaved SNAP-25, even at 400 LD_50_/mL, whereas no interaction was observed in the control cells. Taken together, these results clearly indicated that the immunization process elicited a strong and specific response, thus making the rabbits promising candidates for the isolation of the desired monoclonal antibodies.

### 3.2. Library Construction and Panning

To ensure the highest level of antigen-specific B cells, rabbits were sacrificed 10 days after the last boost [42,43,44,45]. A set of degenerate primers [29] was used to amplify rabbit VH and Vκ sequences from the spleen, bone marrow and peripheral blood cells of each of the two rabbits. VH-and Vκ-amplified genes were then assembled by PCR to obtain combinatorial scFv fragments, which were then inserted into a phagemid vector, resulting in the construction of two large (3 × 10^8^ unique clones) phage display libraries. To maximize the possibility of isolating a variety of antibodies, the two libraries were subjected separately to three enrichment (panning) rounds, using streptavidin plates coated with Bio-P1. An additional enrichment course was carried out with the combination of the two libraries and the inclusion of a competition-binding step, where a synthetic peptide (P3), representing the intact SNAP-25, was diluted into the phage-blocking buffer. Following panning, individual clones from each panning process were screened against adhered specific and non-specific peptides (Bio-P1 and Bio-P2, respectively). Around 50% of the analyzed clones specifically recognized Bio-P1 for both enrichment processes involving the separate libraries. The additional enrichment course, where a negative selection was applied, resulted in only 3% positive clones. Despite the different observed efficiencies, sequence analyses of the selected scFvs indicated that, for each of the three panning processes, only a single unique antibody was isolated (SNAP1, SNAP2 and SNAP3, respectively). The three selected antibodies shared high similarity in their complementarity-determining regions (CDRs), as detailed in Table 1. Overall, the three antibodies had 78% identical residues with an additional 14% similar residues (conservative and semi-conservative homology) in their heavy chains and 87% identity and 8% similarity in their light chains. As the frameworks and CDRs of antibodies SNAP2 and SNAP3 shared the highest similarity, it was logical to assume that both originated from rabbit 1. 

**Figure 3:**
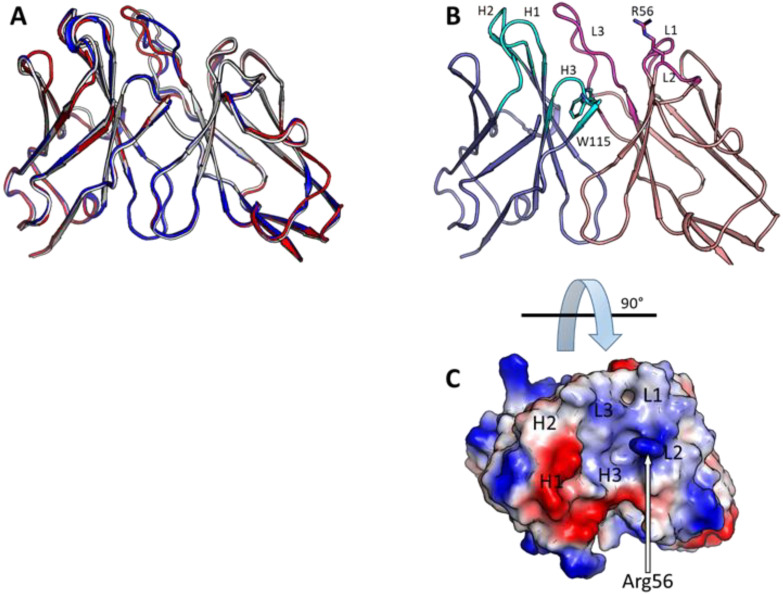
Structure prediction of antibodies. (**A**) Model structures of the three isolated antibodies (scFvs) indicating that all three antibodies are predicted to have very similar structures (red: SNAP1, blue: SNAP2, white: SNAP3). (**B**) Model structure of SNAP1 scFv with light chains colored in pink and heavy chains colored in blue. Rare Trp115 in CDR H3 (cyan sticks) and solvent-exposed Arg56 in CDR L2 (magenta sticks) are shown. (**C**) Electrostatic model of SNAP1 scFv shown indicating that the short CDR H3 creates a groove in the predicted structure.

Another interesting characteristic of the three isolated antibodies was an uncommonly short heavy-chain CDR3 (HCDR3, Table 1). While rabbit CDR3 heavy chains usually contain 12–13 residues [46,47], the BoNT/E-cleaved SNAP-25 antibodies contained 6-residue CDR3s. This feature was unique, as antibodies containing such short HCDR3 were expressed in less than 2.5% of sequenced rabbit antibodies [46,47]. In addition, the apical region of the HCDR3 in rabbits is usually rich in glycine, serine and tyrosine, which were completely absent from these antibodies. Instead, the HCDR3 contained a tryptophan in IMGT-numbered position 115 (Trp115), a bulky aromatic amino acid which is present in less than 2% of rabbit antibody HCDR3s [47]. To gain structural insight into these novel antibodies, we used the AlphaFold2 ab initio structure prediction algorithm-based ColabFold webserver to generate five model structures for each antibody. The structures were ranked by predicted TM (pTM) score, with the highest-ranking model being used for further analysis (Figure 3A). Notably, the top-ranked model for each structure had high pTM scores of 0.917, 0.901, and 0.904 and high mean pLDDT scores of 93.9, 94.4, and 94.2, respectively, suggesting that the models were reliable. The three conformations were quite similar to one another outside of some variation in LCDR3, and in all three antibodies, the model structure shows a deep groove, which may mediate the specific binding to the cleaved SNAP-25. Furthermore, the unique Trp115 amino acid was buried within the core of the light–heavy chain interface where it formed stabilizing hydrophobic interactions with other framework and CDR elements (Figure 3B,C). Furthermore, all three antibodies had a solvent-exposed arginine (Arg56) in the light chain lining the groove, with a particularly deep depression near Arg56. 

### 3.3. Characterization of the Chimeric SNAP1 Antibody

**Figure 4:**
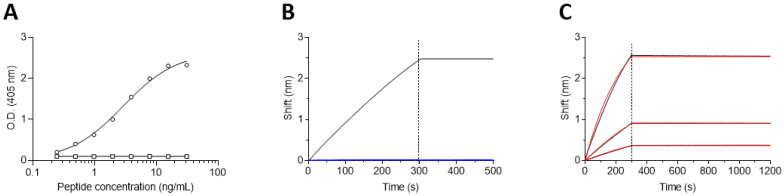
Specificity and affinity of SNAP1. (**A**) Streptavidin-coated ELISA plates were loaded with increasing concentrations of either Bio-P1 (circles) or Bio-P2 (squares) peptides and the binding of SNAP1 antibody was determined. (**B**) Octet Red BLI sensors were loaded with either Bio-P1 (black line) or Bio-P2 (Blue line) peptides. SNAP1 antibody was interacted with the sensors for 300 s, followed by a wash (200 s). (**C**) Bio-P1 peptide was immobilized on a streptavidin-biosensor and reacted for 300 s (association phase) with increasing concentrations of SNAP1 antibody (black lines; from bottom up: 6.7 nM, 20 nM, and 60 nM). The sensors were then immersed in buffer for another 900 s (dissociation phase). Red lines: curve fitting of the 1:1 binding model.

Further characterization of binding and specificity requires a full-length antibody. As the three antibodies exhibited similar activities (determined by ELISA; Data not shown), SNAP1 was reformatted as a scFv-Fc antibody [29]. The specificity of the SNAP1 antibody was first demonstrated using ELISA, with plates coated with increasing concentrations of either the Bio-P1 peptide or Bio-P2 as a negative control. As expected, the antibody bound only the Bio-P1 peptide (representing the cleavage product) without any interaction with the Bio-P2 peptide (Figure 4A). 

The affinity of SNAP1 towards Bio-P1 was next determined using the Octet Red biolayer interferometry (BLI) system. Classical affinity measurements are generally carried out with a sensor-immobilized antibody (ligand) that interacted with an analyte (peptide) in solution [31]. However, the use of small peptides as analytes is not recommended, as they do not induce sufficient interference, thus limiting the accuracy of affinity measurements. We therefore immobilized the biotinylated peptides to the sensors and measured affinity using the antibody as the analyte. In order to minimize avidity effects, the peptides were loaded on the sensor, ensuring low surface density (minimal wavelength shift, nm). To ensure assay specificity, the interaction of the SNAP1 antibody was initially monitored against both the Bio-P1 and Bio-P2 peptides. Indeed, the antibody specifically recognized the immobilized Bio-P1 and did not interact with Bio-P2 (Figure 4B). 

In order to determine SNAP1’s affinity towards cleaved SNAP-25, the Bio-P1 coated sensors were incubated with increasing concentrations of the antibody (the association phase), followed by a wash step (dissociation phase) (Figure 4C). The sensorgrams were fitted with a 1:1 binding model in order to determine the K*_on_* and K*_off_* rates. While the association rate of the antibody could be measured (5.8 × 10^5^ 1/Ms), the dissociation rate was extremely slow (below the Octet Red detection limit, 1 × 10^−7^ s^−1^) and could not be measured, indicating that SNAP1’s affinity is in the sub-pM range.

### 3.4. Binding Characteristics of SNAP1 to the Cleaved SNAP-25 Product

**Figure 5:**
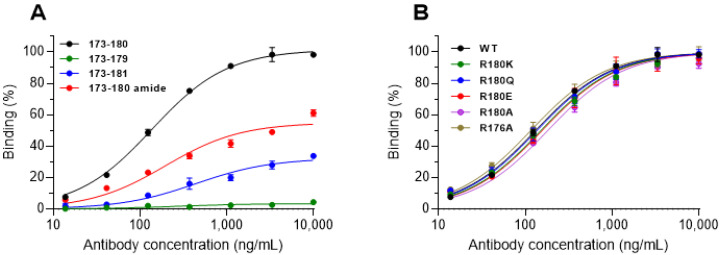
SNAP1 binding to modified SNAP-25 neoepitope peptides. Streptavidin-coated ELISA plates were loaded with (**A**) biotinylated peptides representing either the neoepitope sequence (173–180), a shorter version (173–179), or a longer version (173–181). The modified version (173–180 amide) contained an amide at the end of the peptide instead of a carboxyl group. (**B**) Biotinylated peptides in which the arginines at positions 176 or 180 were substituted with the indicated residues. SNAP1 was added to the bound peptides, and the residual binding was measured. Data are expressed as percent of maximum binding signal obtained with Bio-P1. Points are the mean ± STD of triplicates, fitted by non-linear regression.

The data presented so far, combining SNAP1’s unique HCDR3 with its high affinity toward the peptide representing the BoNT/E SNAP-25-cleaved product, prompted us to further characterize the antibody–peptide interactions. Thus, the SNAP1 antibody was reacted with several versions of the Bio-P1 peptide and the binding was measured. Two groups of peptides were analyzed, one that either shortened, elongated or modified the existing peptide (Figure 5A) and another that substituted the C-terminal arginine with a polar, hydrophobic, negatively charged or shorter side-chain amino acid residue (Figure 5B). Removal of the C-terminal Arg (R180) completely abolished antibody binding to the peptide, while elongation (by an additional isoleucine: I181, the next amino acid in SNAP-25 sequence), resulted in an 80% reduction. Moreover, amidation of the C-terminal end of the peptide, abolishing the negative charge of the carboxyl and effectively mimicking the next peptide bond of the full-length native protein, also significantly decreased SNAP1 binding to the peptide. These results indicated that the negative charge at the end of the chain (in the free peptide or in the cleaved SNAP form) was critical for binding. Furthermore, replacing the C-terminal arginine with alanine, lysine, glutamine or glutamic acid did not affect the binding characteristics, despite the radical substitutions (Figure 5B), and neither had the insertion of a point mutation (R176A) inside the peptide. Thus, one plausible mechanistic explanation is that the antibody specifically recognizes the cleavage site on the neoepitope, potentially through electrostatic interactions to the carboxy-terminus rather than through interactions with the Arg180 sidechain. Although this is quite speculative, the model structures for all three antibodies give a possible structural explanation for this, in which the exposed Arg56 in the light chain provides a positive patch to bind the negatively charged C-terminus of the peptide. 

### 3.5. Selective Detection of BoNT/E Mediated Cleavage of SNAP-25

**Figure 6:**
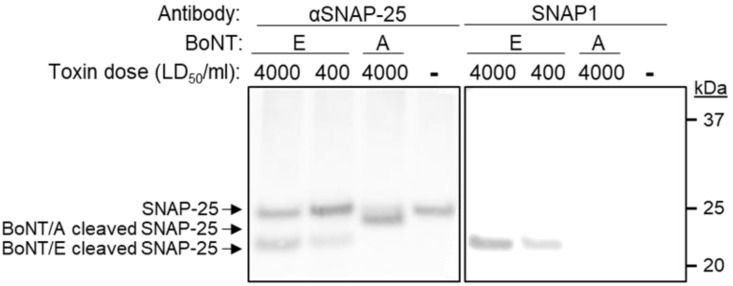
Specific detection of cleaved SNAP-25 neoepitope by SNAP1 antibody. Differentiated SiMa cells were exposed to the indicated doses of either BoNT/A or BoNT/E and lysed 24 h after intoxication. Lysates were subjected to SDS-PAGE and Western-blotted using either anti-SNAP-25 polyclonal antibody or SNAP1.

As the major goal of this study was to develop a method that would enable the selective detection of BoNT/E-mediated cleaved SNAP-25 (SNAP-25_1–180_), we next tested whether the novel SNAP1 antibody was suitable for such an assay. Differentiated SiMa cells were exposed to BoNT/E at a concentration of either 400 or 4000 LD_50_/mL and SNAP-25 and the cell lysates were analyzed by SDS-PAGE. As a positive control, an anti-SNAP-25 antibody directed against the N-terminus protein region was applied, and it demonstrated a similar pattern for the cleavage of SNAP-25 by the toxin (Figure 6). Next, the cell lysates were incubated with SNAP1 antibody and a selective recognition of only the newly formed SNAP-25_1–180_ was indeed achieved. To further demonstrate the specificity of the novel antibody, SiMa cells were exposed to BoNT/A, which cleaves SNAP-25 between residues 197 and 198 (rather than between residues 180 and 181 by BoNT/E). The intoxication process by BoNT/A was verified using the anti-SNAP-25 N-terminus antibody, demonstrating the appearance of the expected cleaved product (Figure 6). However, the BoNT/A cleaved SNAP-25 could not be detected by the SNAP1 antibody, attesting to its high specificity toward the cleaved product of BoNT/E. 

## 4. Discussion

In this work we describe the isolation of three scFv antibodies directed against a peptide that mimics the cleavage product of SNAP-25 following the enzymatic activity of BoNT/E. One of these clones (SNAP1) was further reformatted into a scFv-Fc antibody and was shown to bind the peptide with extremely high affinity and to specifically recognize the product of BoNT/E cleaved SNAP-25 in human neuroblastoma cells.

In the course of this study, we immunized both mice and rabbits, aiming to broaden the subsequent antibody repertoire. The results indicated that the humoral response of the immunized rabbits was both more robust and more specific than that observed in the immunized mice. These results are in accordance with previous reports that rabbits can develop antibodies against unique epitopes on human antigens that are not immunogenic in rodents [48] and elicit strong immune responses against small molecules and haptens, which is rare in rodents [49,50,51,52,53]. These differences may stem from the fact that rabbits belong to the taxonomic order Lagomorpha, which is evolutionary distinct from the order Rodentia, to which mice belong [54].

Another interesting finding was that the three isolated clones originating from two different rabbits shared very high similarity in their sequence and structure. These unique features, including the short HCDR3 and the presence of the Trp amino acid (Trp115) in the HCDR3, most probably contributed to the high affinity and specificity of the SNAP1 antibody. Ig-blast analysis of these three clones revealed that their heavy chains shared a common VH germline origin (IGHV1S69-1). A previous work that performed next-generation sequencing (NGS) of the antibody repertoire in the bone marrow and spleen of a naïve rabbit revealed that this germline is quite rare (0.01% or 0.05% respectively) [46]. However, in the same study, following immunization using a KLH-conjugated 16-mer peptide, the same germline gene was found to comprise 40% of the rabbit’s antibody repertoire [46]. Therefore, this germline may be uniquely fit to bind short peptides. In addition, a study of 235 cDNA clones from human peripheral blood suggested that a smaller CDR3 may create unique binding pockets that allow better interactions between the antigens and CDRs 1 and 2 [55]. It should also be noted that a similar, uncommonly short HCDR3 was observed in a mouse monoclonal antibody that specifically recognized the cleavage site of SNAP-25 by BoNT/A [56]. Interestingly, this antibody contained a 5-residue HCDR3, whereas mouse antibodies most frequently contain 9-residue HCDR3s [47].

We were surprised to find that, despite the fact that the only apparent difference between the intact and cleaved SNAP-25 was the newly exposed Arg180, the side chain of this residue was not critical for antibody recognition and could be replaced by a wide range of other amino acids with different chemical characteristics with no change in affinity. This result supports the notion that the interaction at the carboxy terminus of this newly exposed position occurs via the peptide backbone. We therefore propose the following scenario for SNAP1 mode of action: The specific recognition of the neoepitope depends on the presence of the newly created negative charge of the backbone carboxyl group. In addition, following cleavage, the helical structure of SNAP-25 [57] is disrupted and the exposed neoepitope adopts a unique structure (before or upon interaction with the antibody; induced fit) that fits snugly in the binding pocket of the antibody. Thus, it is suggested that the unique features of the SNAP1 antibody, namely its extremely high affinity and specificity towards the cleaved peptide, are associated with the tight interactions in the unique pocket.

In an effort to dramatically reduce the use of laboratory animals, this antibody was very recently applied in an in vitro SiMa-based assay that measured BoNT/E activity (demonstrating a dose-response relationship between BoNT/E concentrations 250–4000 MsLD_50_/mL and SNAP-25 cleavage), enabling the successful determination of the potency of antitoxin preparations [38].

To summarize, by combining efficient immunization and powerful selection procedures, we successfully isolated a novel, specific, high-affinity antibody against the BoNT/E derived SNAP-25 neoepitope.

## Figures and Tables

**Table 1:** Amino acid sequences of HCDRs and LCDRs.

		Heavy Chain			Light Chain		
Ab	Source	CDR1 ^1^	CDR2	CDR3	CDR1	CDR2	CDR3
SNAP1	Rabbit 2	GIDLSDSA	IYIGSGSA	ARAWDI	ENIYNN	RAS	QSSYVGTHVNYGND
SNAP2	Rabbit 1	GINLSSSA	IYAGSGNT	ATAWDI	QSIGSN	RAS	QSSYVGTHINYGNG
SNAP3	Rabbits 1 + 2	GIDFSDNA	TYVGSGRT	ARTWDI	ENIYNN	RAS	QSSYVGTTINYGNG

^1^ CDRs are determined according to International ImMunoGeneTics (IMGT) annotation.

## Data Availability

The datasets generated for this study are available on request to the corresponding author.

## Supplementary Materials

The following are available online at https://www.mdpi.com/article/10.3390/antib11010021/s1, Figure S1: Densitometry analysis of SNAP-25 western blot. Differentiated SiMa cells were exposed to 0 or 1000 LD_50_/ml of BoNT/E (- BoNT/E or + BoNT/E) and lysed 24 h after intoxication. Lysates were subjected to SDS-PAGE and Western-blotted using anti-SNAP-25 polyclonal antibody (lower panel). Band signals were analyzed for densitometry (upper panel) using ImageJ (version 1.51) and values are depicted within each peak.

## References

[1] Paddle B.M. (2003). Therapy and prophylaxis of inhaled biological toxins. J. Appl. Toxicol..

[2] Pirazzini M., Rossetto O., Eleopra R., Montecucco C. (2017). Botulinum Neurotoxins: Biology, Pharmacology, and Toxicology. Pharmacol. Rev..

[3] Lacy D.B., Stevens R.C. (1999). Sequence homology and structural analysis of the clostridial neurotoxins. J. Mol. Biol..

[4] Simpson L.L. (2004). Identification of the major steps in botulinum toxin action. Annu. Rev. Pharmacol. Toxicol..

[5] Schiavo G., Matteoli M., Montecucco C. (2000). Neurotoxins affecting neuroexocytosis. Physiol. Rev..

[6] Montal M. (2010). Botulinum Neurotoxin: A Marvel of Protein Design. Annu. Rev. Biochem..

[7] Rusnak J.M., Smith L.A. (2009). Botulinum neurotoxin vaccines: Past history and recent developments. Hum. Vaccines.

[8] Smith L.A., Rusnak J.M. (2007). Botulinum Neurotoxin Vaccines: Past, Present, and Future. Crit. Rev. Immunol..

[9] Binz T., Blasi J., Yamasaki S., Baumeister A., Link E., Südhof T.C., Jahn R., Niemann H. (1994). Proteolysis of SNAP-25 by types E and A botulinal neurotoxins. J. Biol. Chem..

[10] Schiavo G., Santucci A., Dasgupta B.R., Mehta P.P., Jontes J., Benfenati F., Wilson M.C., Montecucco C. (1993). Botulinum neurotoxins serotypes A and E cleave SNAP-25 at distinct COOH-terminal peptide bonds. FEBS Lett..

[11] Wang J., Meng J., Lawrence G.W., Zurawski T.H., Sasse A., Bodeker M.O., Gilmore M.A., Fernández-Salas E., Francis J., Steward L.E. (2008). Novel chimeras of botulinum neurotoxins A and E unveil contributions from the binding, translocation, and protease domains to their functional characteristics. J. Biol. Chem..

[12] Dembek Z.F., Smith L.A., Rusnak J.M. (2007). Botulism: Cause, effects, diagnosis, clinical and laboratory identification, and treatment modalities. Disaster Med. Public Health Prep..

[13] MMWR (2010). Investigational heptavalent botulinum antitoxin (HBAT) to replace licensed botulinum antitoxin AB and investigational botulinum antitoxin E. MMWR Morb. Mortal. Wkly. Rep..

[14] Infant Botulism Treatment and Prevention Program. http://www.infantbotulism.org.

[15] European Directorate for the Quality of Medicines and Healthcare (2019). Botulinum Toxin Type A/Type B for Injection.

[16] Torgeman A., Schwartz A., Diamant E., Baruchi T., Dor E., Ben David A., Pass A., Barnea A., Tal A., Rosner A. (2018). Studying the differential efficacy of postsymptom antitoxin treatment in type A versus type B botulism using a rabbit spirometry model. Dis. Model. Mech.

[17] Behrensdorf-Nicol H.A., Wild E., Bonifas U., Klimek J., Hanschmann K.M., Krämer B., Kegel B. (2018). In vitro potency determination of botulinum neurotoxin serotype A based on its receptor-binding and proteolytic characteristics. Toxicol. Int. J. Publ. Assoc. BIBRA.

[18] Dressler D., Dirnberger G. (2001). Botulinum toxin antibody testing: Comparison between the immunoprecipitation assay and the mouse diaphragm assay. Eur. Neurol..

[19] Hanna P.A., Jankovic J. (1998). Mouse bioassay versus Western blot assay for botulinum toxin antibodies: Correlation with clinical response. Neurology.

[20] Lindsey C.Y., Smith L.A., West M.W., Boles J.W., Brown J.E. (2003). Evaluation of a botulinum fragment C-based ELISA for measuring the humoral immune response in primates. Biologicals.

[21] Palace J., Nairne A., Hyman N., Doherty T.V., Vincent A. (1998). A radioimmuno-precipitation assay for antibodies to botulinum A. Neurology.

[22] Rosen O., Ozeri E., Barnea A., David A.B., Zichel R. (2016). Development of an Innovative in Vitro Potency Assay for Anti-Botulinum Antitoxins. Toxins.

[23] Wild E., Bonifas U., Klimek J., Trösemeier J.H., Krämer B., Kegel B., Behrensdorf-Nicol H.A. (2016). In vitro potency determination of botulinum neurotoxin B based on its receptor-binding and proteolytic characteristics. Toxicol. Int. J. Publ. Assoc. BIBRA.

[24] Fernandez-Salas E., Wang J., Molina Y., Nelson J.B., Jacky B.P., Aoki K.R. (2012). Botulinum neurotoxin serotype A specific cell-based potency assay to replace the mouse bioassay. PLoS ONE.

[25] Torgeman A., Diamant E., Levin L., Ben David A., Epstein E., Girshengorn M., Mazor O., Rosenfeld R., Zichel R. (2017). An in vitro cell-based potency assay for pharmaceutical type A botulinum antitoxins. Vaccine.

[26] Bak N., Rajagopal S., Stickings P., Sesardic D. (2017). SiMa Cells for a Serotype Specific and Sensitive Cell-Based Neutralization Test for Botulinum Toxin A and E. Toxins.

[27] Leveque C., Ferracci G. (2015). An optical biosensor assay for rapid dual detection of Botulinum neurotoxins A and E. Sci. Rep..

[28] von Berg L., Stern D., Pauly D., Mahrhold S., Weisemann J., Jentsch L., Hansbauer E.M., Muller C., Avondet M.A., Rummel A. (2019). Functional detection of botulinum neurotoxin serotypes A to F by monoclonal neoepitope-specific antibodies and suspension array technology. Sci. Rep..

[29] Mechaly A., Alcalay R., Noy-Porat T., Epstein E., Gal Y., Mazor O. (2018). Novel Phage Display-Derived Anti-Abrin Antibodies Confer Post-Exposure Protection against Abrin Intoxication. Toxins.

[30] Mechaly A., Elia U., Alcalay R., Cohen H., Epstein E., Cohen O., Mazor O. (2019). Inhibition of Francisella tularensis phagocytosis using a novel anti-LPS scFv antibody fragment. Sci. Rep..

[31] Noy-Porat T., Rosenfeld R., Ariel N., Epstein E., Alcalay R., Zvi A., Kronman C., Ordentlich A., Mazor O. (2016). Isolation of Anti-Ricin Protective Antibodies Exhibiting High Affinity from Immunized Non-Human Primates. Toxins.

[32] Azriel-Rosenfeld R., Valensi M., Benhar I. (2004). A human synthetic combinatorial library of arrayable single-chain antibodies based on shuffling in vivo formed CDRs into general framework regions. J. Mol. Biol..

[33] Rosenfeld R., Marcus H., Ben-Arie E., Lachmi B., Mechaly A., Reuveny S., Gat O., Mazor O., Ordentlich A. (2009). Isolation and Chimerization of a Highly Neutralizing Antibody conferring Passive Protection against Lethal *B. anthracis* Infection. PLoS ONE.

[34] Bujak E., Matasci M. (2014). Reformatting of scFv Antibodies into the scFv-Fc Format and Their Downstream Purification.

[35] Noy-Porat T., Alcalay R., Mechaly A., Peretz E., Makdasi E., Rosenfeld R., Mazor O. (2021). Characterization of antibody-antigen interactions using biolayer interferometry. STAR Protoc..

[36] Whelan S.M., Elmore M.J., Bodsworth N.J., Atkinson T., Minton N.P. (1992). The complete amino acid sequence of the Clostridium botulinum type-E neurotoxin, derived by nucleotide-sequence analysis of the encoding gene. Eur. J. Biochem..

[37] Binz T., Kurazono H., Wille M., Frevert J., Wernars K., Niemann H. (1990). The complete sequence of botulinum neurotoxin type A and comparison with other clostridial neurotoxins. J. Biol. Chem..

[38] Diamant E., Torgeman A., Epstein E., Mechaly A., David A.B., Levin L., Schwartz A., Dor E., Girshengorn M., Barnea A. (2022). A cell-based alternative to the mouse potency assay for pharmaceutical type E botulinum antitoxins. ALTEX.

[39] Mirdita M., Schütze K., Moriwaki Y., Heo L., Ovchinnikov S., Steinegger M. (2021). ColabFold—Making protein folding accessible to all. bioRxiv.

[40] Jumper J., Evans R., Pritzel A., Green T., Figurnov M., Ronneberger O., Tunyasuvunakool K., Bates R., Zidek A., Potapenko A. (2021). Highly accurate protein structure prediction with AlphaFold. Nature.

[41] Evans R., O’Neill M., Pritzel A., Antropova N., Senior A., Green T., Žídek A., Bates R., Blackwell S., Yim J. (2021). Protein complex prediction with AlphaFold-Multimer. bioRxiv.

[42] Wrammert J., Smith K., Miller J., Langley W.A., Kokko K., Larsen C., Zheng N., Mays I., Garman L., Helms C. (2008). Rapid cloning of high-affinity human monoclonal antibodies against influenza virus. Nature.

[43] Greenfield E.A. (2020). Standard Immunization of Rabbits. Cold Spring Harb. Protoc..

[44] Overduin L.A., van Dongen J.J.M., Visser L.G. (2019). The Cellular Immune Response to Rabies Vaccination: A Systematic Review. Vaccines.

[45] Webster R.G. (1968). The immune response to influenza virus. II. Effect of the route and schedule of vaccination on the quantity and avidity of antibodies. Immunology.

[46] Kodangattil S., Huard C., Ross C., Li J., Gao H., Mascioni A., Hodawadekar S., Naik S., Min-debartolo J., Visintin A. (2014). The functional repertoire of rabbit antibodies and antibody discovery via next-generation sequencing. mAbs.

[47] Lavinder J.J., Hoi K.H., Reddy S.T., Wine Y., Georgiou G. (2014). Systematic characterization and comparative analysis of the rabbit immunoglobulin repertoire. PLoS ONE.

[48] Rief N., Waschow C., Nastainczyk W., Montenarh M., Gotz C. (1998). Production and characterization of a rabbit monoclonal antibody against human CDC25C phosphatase. Hybridoma.

[49] Feng L., Wang X., Jin H. (2011). Rabbit monoclonal antibody: Potential application in cancer therapy. Am. J. Transl. Res..

[50] Li Y., Cockburn W., Kilpatrick J.B., Whitelam G.C. (2000). High affinity ScFvs from a single rabbit immunized with multiple haptens. Biochem. Biophys. Res. Commun..

[51] Liu N., Han Z., Lu L., Wang L., Ni G., Zhao Z., Wu A., Zheng X. (2013). Development of a new rabbit monoclonal antibody and its based competitive indirect enzyme-linked immunosorbent assay for rapid detection of sulfonamides. J. Sci. Food Agric..

[52] Liu N., Zhao Z., Tan Y., Lu L., Wang L., Liao Y., Beloglazova N., De Saeger S., Zheng X., Wu A. (2016). Simultaneous Raising of Rabbit Monoclonal Antibodies to Fluoroquinolones with Diverse Recognition Functionalities via Single Mixture Immunization. Anal. Chem..

[53] Zhu X., Kriegel A.M., Boustany C.A., Blake D.A. (2011). Single-chain variable fragment (scFv) antibodies optimized for environmental analysis of uranium. Anal. Chem..

[54] Miller W., Rosenbloom K., Hardison R.C., Hou M., Taylor J., Raney B., Burhans R., King D.C., Baertsch R., Blankenberg D. (2007). 28-way vertebrate alignment and conservation track in the UCSC Genome Browser. Genome Res..

[55] Rosner K., Winter D.B., Tarone R.E., Skovgaard G.L., Bohr V.A., Gearhart P.J. (2001). Third complementarity-determining region of mutated VH immunoglobulin genes contains shorter V, D, J, P, and N components than non-mutated genes. Immunology.

[56] Fernandes-Salas E., Wang J., Kei R. (2012). Immuno-Based Botulinum Toxin Serotype A Activity Assays. U.S. Patent.

[57] Chen X., Tomchick D.R., Kovrigin E., Arac D., Machius M., Sudhof T.C., Rizo J. (2002). Three-dimensional structure of the complexin/SNARE complex. Neuron.

