# Supplementary material for: Highly Specific Monoclonal Antibody Targeting the Botulinum Neurotoxin Type E Exposed SNAP-25 Neoepitope
Source: Antibodies (Basel). 2022 Mar 16;11(1):21. doi: 10.3390/antib11010021 (PMC8944829; doi:10.3390/antib11010021)
Supplement: Supplementary file 1 [file antibodies-11-00021-s001.zip › antibodies-1602123-supplementary.pdf]

Supplementary Materials:

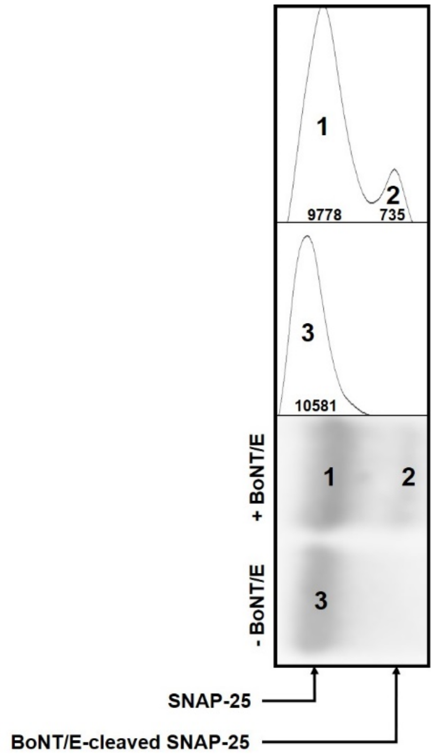

Figure S1. Densitometry analysis of SNAP-25 western blot. Differentiated SiMa cells were exposed to 0 or 1000 LD_{50}/ml of BoNT/E (- BoNT/E or + BoNT/E) and lysed 24 hours after intoxication. Lysates were subjected to SDS-PAGE and Western-blotted using anti-SNAP-25 polyclonal antibody (lower panel). Band signals were analyzed for densitometry (upper panel) using ImageJ (version 1.51) and values are depicted within each peak..
